# Verschiedene immunologische Typen der CRSwNP im Kontext der neuen Europäischen EAACI-Nomenklatur: Teil 2: Hypersensitivitätsreaktionen vom Typ V (epitheliale Barrieredefekte)

**DOI:** 10.1007/s00106-025-01705-1

**Published:** 2025-12-29

**Authors:** L. Klimek, S. Becker, B. Haxel, M. Cuevas, P. Huber, A. Chaker, O. Pfaar, M. Laudien, C. Beutner, J. Hagemann, U. Förster-Ruhrmann, H. Olze, B. P. Ernst, A. Beule, C. Rudack, A. S. Hoffmann, C. Betz, M. Gröger

**Affiliations:** 1 Zentrum für Rhinologie und Allergologie, Wiesbaden, Deutschland; 2 Klinik für Hals‑, Nasen- und Ohrenheilkunde, Universitätsmedizin Tübingen, Tübingen, Deutschland; 3 Klinik für Hals‑, Nasen- und Ohrenheilkunde, Universitätsmedizin Mainz, Mainz, Deutschland; 4 Klinik und Poliklinik für HNO-Heilkunde, Medizinische Fakultät und Universitätsklinikum Carl Gustav Carus, TU Dresden, Dresden, Deutschland; 5 Klinik und Poliklinik für Hals-Nasen- Ohrenheilkunde, Klinikum der Ludwig-Maximilians-Universität München, München, Deutschland; 6 TUM School of Medicine and Health, Klinikum rechts der Isar, HNO-Klinik und Zentrum für Allergie und Umwelt, Technische Universität München, München, Deutschland; 7 Klinik für Hals‑, Nasen- und Ohrenheilkunde, Universitätsklinikum Gießen und Marburg GmbH, Standort Marburg, Marburg, Deutschland; 8 Klinik für Hals‑, Nasen- und Ohrenheilkunde, Universitätsklinikum Kiel, Kiel, Deutschland; 9 Klinik für Dermatologie, Venerologie und Allergologie, Universitätsmedizin Göttingen, Göttingen, Deutschland; 10 Charité-Universitätsmedizin Berlin, Berlin, Deutschland; 11 Klinik für Hals‑, Nasen- und Ohrenheilkunde, Universitätsmedizin Frankfurt/M., Frankfurt/M., Deutschland; 12 Klinik für Hals‑, Nasen- und Ohrenheilkunde, Universitätsklinikum Münster, Münster, Deutschland; 13 Klinik für Hals‑, Nasen- und Ohrenheilkunde, Universitätsklinikum Hamburg-Eppendorf, Hamburg, Deutschland

**Keywords:** Umweltbedingte Erkrankungen, Rhinosinusitis, Hypersensitivität, Nasenpolypen, Biologika, Environmental illness, Rhinosinusitis, Hypersensitivity, Nasal polyps, Biologics

## Abstract

**Hintergrund:**

Die chronische Rhinosinusitis (CRS) weist in Europa und den USA eine Prävalenz von bis zu 11 % auf und gehört somit zu den häufigsten chronischen Erkrankungen überhaupt. Die Klassifizierung nach immunologischen Endotypen findet immer mehr Eingang in die Krankheitsdefinition, vor allem bei der chronischen Rhinosinusitis mit Nasenpolypen (CRSwNP). Hierbei spielen genetische und epigenetische Veränderungen im mukosalen Immunsystem eine wichtige Rolle. Die Identifizierung von Endotypen kann dazu beitragen, die Heterogenität innerhalb der Erkrankung zu verstehen und personalisierte Behandlungsansätze zu entwickeln. Im Teil 1 dieser Publikation hatten wir die immunologischen Klassifizierungen der Hypersensitivitätsreaktionen vom Typ IV dargestellt (T1-, T2-, und T3-Endotypen).

**Methodik:**

Die Europäische Akademie für Allergie und klinische Immunologie (EAACI) hat in einem kürzlich erschienenen Positionspapier eine Aktualisierung der Nomenklatur für immunologische Überempfindlichkeitsreaktionen auf der Basis von neun verschiedenen immunologischen Reaktionstypen vorgestellt. Die ursprünglich von Coombs und Gell klassifizierten antikörpervermittelten Reaktionen vom Typ I, Typ II und Typ III wurden erweitert und detailliert beschrieben. Epitheliale Barrieredefekte werden als Typ V dieser Hypersensitivitätsreaktionen definiert und werden im hier vorliegenden Teil 2 dieser Publikationsreihe erörtert.

**Ergebnisse:**

Der Typ V geht mit einer Beeinträchtigung der epithelialen Barrierefunktion einher und führt zu einer fehlerhaften und andauernden Aktivierung des mukosalen Immunsystems und folglich zu chronischen Entzündungen. Neben dem Defekt der Epithelbarriere ist hieran wesentlich die mikrobielle Dysbiose mit Dysregulation der Immunantwort beteiligt, einschließlich einer umfassenden Aktivierung von Th1, Th2 und Th17 in Verbindung mit dem Verlust von regulatorischen T‑ und B‑Zellen (Treg) und (Breg). Hinzu kommen die Bildung von Serum-(s)IgE für Inhalations- oder Nahrungsmittelallergene, die Aktivierung von Makrophagen (Mφ) und die Freisetzung von proinflammatorischen Zytokinen, Chemokinen und Entzündungsmediatoren (Histamin, Leukotriene). Der Verlust der Barrierefunktion kann auf Defekte in mehreren wesentlichen Komponenten zurückzuführen sein, darunter Strukturelemente, Tight-Junction-Proteine, schützende Antiproteasen, die Expression antimikrobieller Proteine, der Transport von Ionen, Protonen, Wasser oder antimikrobiellen Stoffen und andere Mechanismen. Mit dem Verlust der Barriere verbunden ist die Aktivierung sensorischer Nervenfasern in der Schleimhaut, die zur Entwicklung entzündlicher Symptome beitragen.

**Schlussfolgerung:**

Bei Patienten mit CRSwNP können immunologische Hyperreaktivitätsreaktionen unterschiedlicher Typen – insbesondere der Typ-IV-, Typ-V- und Typ-VI-Hypersensitivität – sowohl isoliert als auch in Kombination auftreten. Während Typ-IV- und Typ-VI-Reaktionen maßgeblich zur zellulären Entzündungsantwort und chronischen Persistenz beitragen, gewinnen Typ-V-Reaktionen, die durch fehlgeleitete Rezeptorsignalisierungen charakterisiert sind, zunehmend an Bedeutung im Kontext epithelialer Barrieredefekte und gestörter mukosaler Regeneration. Ziel des vorliegenden zweiten Teils dieser Übersichtsarbeit ist es, die Mechanismen der Typ-V-Hypersensitivitätsreaktionen darzustellen und deren Implikationen für eine erweiterte Diagnostik und therapeutische Ansätze bei CRSwNP zu diskutieren.

## Abstract (en)

**Background:**

Chronic rhinosinusitis (CRS) has a prevalence of up to 11% in Europe and the United States, making it one of the most common chronic diseases. Classification according to immunological endotypes is increasingly being incorporated into disease definitions, particularly for chronic rhinosinusitis with nasal polyps (CRSwNP). Genetic and epigenetic alterations within the mucosal immune system play an important role in this context. Identification of endotypes may help to elucidate disease heterogeneity and guide development of personalized therapeutic approaches. In part 1 of this publication series, we presented the immunological classification of type IV hypersensitivity reactions (T1, T2, and T3 endotypes).

**Methods:**

The European Academy of Allergy and Clinical Immunology (EAACI) recently published a position paper introducing the updated nomenclature for immunological hypersensitivity reactions based on nine distinct immunological reaction types. The antibody-mediated reactions originally classified by Coombs and Gell as type I, type II, and type III have been expanded and described in greater detail. Epithelial barrier defects are defined as type V hypersensitivity reactions and are the focus of part 2 of this publication series.

**Results:**

Type V hypersensitivity is characterized by impairment of epithelial barrier function, resulting in aberrant and persistent activation of the mucosal immune system and, consequently, chronic inflammation. In addition to barrier dysfunction, microbial dysbiosis and dysregulation of immune responses play a major role, including broad activation of Th1, Th2, and Th17 pathways, accompanied by a loss of regulatory T and B cells (Treg, Breg). Further mechanisms involve the production of serum (s)IgE against inhalant or food allergens; activation of macrophages (Mφ); and release of proinflammatory cytokines, chemokines, and inflammatory mediators such as histamine and leukotrienes. Loss of epithelial barrier integrity may result from defects in several key components, including structural elements; tight junction proteins; protective antiproteases; expression of antimicrobial proteins; and altered transport of ions, protons, water, or antimicrobial substances. Disruption of the epithelial barrier is also associated with activation of sensory nerve fibers within the mucosa, thereby contributing to the development of inflammatory symptoms.

**Conclusion:**

In patients with CRSwNP, immunological hyperreactivity reactions of various types—particularly type IV, type V, and type VI hypersensitivity—may occur in either isolation or combination. While type IV and type VI reactions primarily contribute to the cellular inflammatory response and chronic persistence, type V reactions, characterized by dysregulated receptor signaling, are increasingly recognized as clinically relevant in the context of epithelial barrier defects and impaired mucosal regeneration. The aim of this second part of the review is to elucidate the mechanisms underlying type V hypersensitivity reactions and to discuss their implications for extended diagnostic and therapeutic approaches in CRSwNP.

Die chronische Rhinosinusitis (CRS) weist in Europa und den USA eine Prävalenz von bis zu 11 % auf und gehört somit zu den häufigsten chronischen Erkrankungen überhaupt [21]. Die Diagnose einer chronischen Rhinosinusitis (CRS) basiert gemäß den aktuellen Kriterien des European Position Paper on Rhinosinusitis and Nasal Polyps 2020 (EPOS-2020) und der derzeit noch aktuellen deutschen S2k-Leitlinie Rhinosinusitis (AWMF 017-049, AWMF 053-012) auf dem Nachweis einer Entzündung der Nase und der Nasennebenhöhlen, die durch mindestens zwei Symptome gekennzeichnet ist, von denen eines entweder eine nasale Obstruktion/Blockade/Kongestion oder eine nasale Sekretion (anterior oder posterior, „postnasal drip“) sein muss. Begleitend können Gesichts- bzw. Kopfschmerz/-druck und/oder eine Riechminderung bzw. ein Riechverlust (Hyposmie/Anosmie) auftreten. Zusätzlich muss mindestens eines der folgenden objektiven Kriterien vorliegen: endoskopische Zeichen wie nasale Polypen, mukopurulente Sekretion oder Schleimhautschwellung bzw. -obstruktion im mittleren Nasengang und/oder bildgebender Nachweis (CT) von Schleimhautveränderungen innerhalb des Ostiomeatal-Komplexes und/oder der Nasennebenhöhlen. Die Leitlinie unterscheidet weiterhin zwischen akuter, rezidivierend akuter und chronischer Rhinosinusitis, wobei Letztere definitionsgemäß über mehr als 12 Wochen anhält.

Unterschieden wird zwischen einer CRS mit (CRSwNP) und ohne Polypen (CRSsNP). Die CRSwNP ist mit einer Prävalenz von bis zu 4 % deutlich seltener als die CRSsNP, weist jedoch meist einen schwereren Krankheitsverlauf auf [21]. CRSwNP und CRSsNP sind keine einheitlichen Krankheitsbilder, da innerhalb dieser Phänotypen verschiedene Pathomechanismen existieren, die zu unterschiedlichen inflammatorischen Reaktionen der Mukosa führen, welche als Endotypen bezeichnet werden [47]. Das Verständnis der hier dargelegten pathophysiologischen Grundlagen soll in der Zukunft eine präzise, individualisierte Therapie ermöglichen.

Bei der CRSwNP gelten Inhalationsallergien zwar nicht als primärer Pathomechanismus [8, 18, 31, 64], können jedoch als klinisch relevante Komorbidität auftreten und den Krankheitsverlauf wesentlich beeinflussen. In einer britischen populationsbasierten Studie wurde beispielsweise bei rund 31 % der Patienten mit CRSwNP eine Sensibilisierung gegenüber Aeroallergenen nachgewiesen, wobei insbesondere Hausstaubmilbenallergien häufiger als bei CRSsNP vorkamen [45]. Bei der CRSsNP können Allergien vor allem auf perenniale Antigene wie z. B. Milbenantigene eine ursächliche Rolle spielen, weshalb entsprechende Therapieoptionen bedacht und angewendet werden sollten [3–6, 11, 27, 28, 37, 40, 51, 56, 59]. Vielmehr sind bei der CRSwNP fehlgeleitete immunologische Hyperreaktivitätsreaktionen in der Mukosa der Nasennebenhöhlen entscheidend, die in diesem Beitrag näher beleuchtet werden [2, 43, 44, 50].

## Methodik

Der vorliegenden Publikation liegt ein Positionspapier der europäischen Akademie für Allergie und klinische Immunologie (EAACI) zugrunde, in dem eine modernisierte und erweiterte Klassifikation von Hypersensitivitätsreaktionen vorgestellt wird [23]. Diese wurde inzwischen auch auf das deutsche Gesundheitssystem angepasst [22, 24, 25]. Die Bezeichnung „Überempfindlichkeit“ wurde erstmals 1963 von Robin Coombs und Philip George Houthem Gell eingeführt. Überempfindlichkeit bezieht sich auf eine unerwünschte, unangenehme oder schädliche Reaktion, die aus einer Überreaktion der adaptiven Immunantwort resultiert. Sie umfasst sowohl Allergien, die durch äußere Reize ausgelöst werden, als auch Autoimmunität, die auf intrinsische Reize zurückzuführen ist. Typischerweise setzen Überempfindlichkeitsreaktionen einen vorsensibilisierten (Immun‑)Zustand des Organismus voraus (sekundäre Immunantwort). Nach Coombs und Gell wurden Überempfindlichkeitsreaktionen in vier Typen eingeteilt: Typ I: unmittelbar (IgE-vermittelt), Typ II: zytotoxisch (antikörper- und Fc-Rezeptor-vermittelt, zellulär), Typ III: immunkomplexvermittelt, und Typ IV: verzögert (T-Zell-vermittelt) [9, 22–25].

Zusätzlich hierzu führten neue Erkenntnisse zur Definition einer Hypersensitivitätsreaktion durch eine epitheliale Barrierestörung (Typ V), die auch bei der CRS existiert und Gegenstand dieser Übersichtsarbeit ist.

## Hypersensitivitätsreaktion vom Typ V – Defekt der epithelialen Barriere

**Abb. 1:**
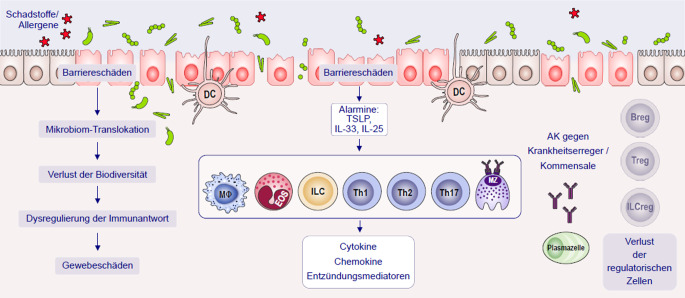
Mechanismen der Typ-V-Überempfindlichkeit zeigen sich bei ganz unterschiedlichen Erkrankungen wie CRS, Asthma, AR/ARC, AD, FPIES, EoE und Zöliakie. Der Defekt der Epithelbarriere und die mikrobielle Dysbiose führen zu einer Dysregulation der Immunantwort, einschließlich einer umfassenden Aktivierung von Th1, Th2 und Th17 in Verbindung mit dem Verlust von regulatorischen T‑ (Treg) und B‑Zellen (Breg). Hinzu kommen die Bildung von Serum-(s)IgE für Inhalations- oder Nahrungsmittelallergene, die Aktivierung von Makrophagen (Mφ) und die Freisetzung von proinflammatorischen Zytokinen, Chemokinen und Entzündungsmediatoren (Histamin, Leukotriene). Diese Abfolge von Ereignissen führt schließlich zu Gewebeschäden, wie sie bei CRS, Asthma, AR/ARC, AD, FPIES, EoE und Zöliakie auftreten können. Die Immunreaktion auf opportunistische Krankheitserreger und Kommensalen, z. B. S. aureus, führt zur Produktion von Antikörpern gegen sie. *AD* atopische Dermatitis; *AR* allergische Rhinitis; *ARC* allergische Rhinokonjunktivitis; *B* B-Lymphozyt; *Breg* regulatorische B‑Zellen; *CRS* chronische Rhinosinusitis; *DC* dendritische Zelle; *Eos* eosinophil; *EoE* eosinophile Ösophagitis; *IL* Interleukin; *ILC* angeborene lymphatische Zelle; *ILCreg* ILC-Regulatorzellen; *MZ* Mastzelle; *Mφ* Makrophage; *FPIES* nahrungsproteininduziertes Enterokolitis-Syndrom; *sIgE* allergenspezifische Immunglobuline Klasse E; *Th1/2/17* T-Helfer-Lymphozyten Typ 1/2/17; *Treg* regulatorische T‑Zellen; *TSLP* thymisches stromales Lymphopoietin

In den letzten Jahren wurden erhebliche Fortschritte beim Verständnis der verschiedenen Phänotypen und Endotypen von Entzündungskrankheiten der Schleimhäute und der Haut wie chronische allergische Rhinitis (AR), allergische Rhinokonjunktivitis (ARC), CRS, atopische Dermatitis (AD), Asthma oder das nahrungsproteininduzierte Enterokolitis-Syndrom (FPIES), die eosinophile Ösophagitis (EoE) und Zöliakie erzielt. Dabei zeigte sich, dass es sich bei diesen Erkrankungen nicht um homogene Krankheiten handelt, sondern um eine Konstellation von Symptomen, die auf unterschiedliche pathologische Mechanismen zurückzuführen sein können [1]. In einigen Fällen ist der Entzündungsprozess auf eine veränderte Barrierefunktion der Haut oder Schleimhaut zurückzuführen und nicht auf eine primäre Dysregulation des Immunsystems [49]. Die Beeinträchtigung der epithelialen Barrierefunktion führt zu einer fehlerhaften und andauernden Aktivierung des mukosalen Immunsystems und führt in der Folge zu chronischen Entzündungen. Der Verlust der Barrierefunktion kann auf Defekte in mehreren wesentlichen Komponenten zurückzuführen sein, darunter Strukturelemente des Stratum corneum in der Haut, Tight-Junction-Proteine in Haut und Schleimhäuten, schützende Antiproteasen, die Expression antimikrobieller Produkte, den Transport von Ionen, Protonen, Wasser oder antimikrobiellen Stoffen und andere Mechanismen. Mit dem Verlust der Barriere ebenfalls verbunden ist die Aktivierung sensorischer Nervenfasern in der Schleimhaut, die zur Entwicklung entzündlicher Symptome beitragen (Abb. 1; [13]).

Mutationen des Filaggrins, eines keratinbindenden Proteins, das für die epidermale Homöostase wichtig ist, könnte für atopische Dermatitis prädisponieren, und zwar bei Personen mit und ohne Immunglobulin-E(IgE)-Sensibilisierung gegenüber Allergenen. Es wird spekuliert, dass Filaggrin-Mutationen eine ähnliche Wirkung auf die Schleimhäute haben und zu Krankheiten wie Asthma prädisponieren könnten [42, 52]. Die Beeinträchtigung der epithelialen Barriere kann auch durch Entzündungsphänomene verursacht werden. Es hat sich gezeigt, dass T‑Helfer-Zellen vom Typ 2 (Th2-Zellen) und von angeborenen lymphoiden Zellen (ILC2) stammendes Interleukin (IL)-13 die epithelialen „tight junctions“ deutlich stören [54]. Die Aktivierung und Gewebemigration von ILC2- und Th2-Zellen hängt stark von den aus dem Epithel stammenden Alarminen ab, insbesondere IL-33, IL-25 und thymisches stromales Lymphopoietin (TSLP) [20]. Dabei könnte die Proteaseaktivität einiger Aeroallergene für die Aktivierung der Epithelzellen der Atemwege, die Freisetzung von IL-33, die Stimulierung von ILC2, die Produktion von IL-13 und die letztendliche Unterbrechung der epithelialen „tight junctions“ verantwortlich sein [53]. Diese verketteten Phänomene können auch ohne Lymphozyten oder Aktivierung der adaptiven Immunität auftreten [67]. Es hat sich gezeigt, dass die „tight junctions“ in der Schleimhaut der Atemwege von Patienten mit allergischer Rhinitis und Asthma ebenfalls gestört sind. Dies zeigt, dass der Barrieredefekt auch bei der klassischen Typ-IVb-Hypersensibilität auftreten kann. Somit besteht offensichtlich eine komplexe Beziehung zwischen verschiedenen Überempfindlichkeitsmechanismen in Haut und Schleimhaut: Die epitheliale Integrität kann durch intrinsische Defekte gedämpft werden, aber auch Entzündungsphänomene können eine Beeinträchtigung der Barrierefunktion verursachen, die das Immunsystem weiter aktiviert.

Die direkte Beteiligung von Umweltfaktoren, die die Epithelbarrieren direkt stören, wurde kürzlich in mehreren Modellen und in menschlichem Gewebe als wichtiger Faktor der Typ-V-Überempfindlichkeit nachgewiesen [48]. Die Gesamtheit aller Umweltfaktoren, denen ein Mensch im Laufe seines Lebens ausgesetzt ist und die seine Gesundheit beeinflussen können, wird als Exposom bezeichnet. Die direkte Exposition gegenüber Luftschadstoffen, Chemikalien und anderen Umweltfaktoren im Exposom kann die Epithelbarrieren stören und das Mikrobiom und das Immunsystem beeinträchtigen. Es ist bekannt, dass viele der chemischen Stoffe, die in gängigen Konsumgütern (wie Zahnpasta, Shampoo, Reinigungsmitteln und verarbeiteten Lebensmitteln) enthalten sind, diese kritischen Barrieren schädigen und die Durchlässigkeit für Bakterien, Toxine, Schadstoffe und Allergene erhöhen [39]. Wenn die Epithelbarrieren gestört (oder „undicht“) sind, können Substanzen und Mikroben in tiefer gelegenes Gewebe eindringen, wo sie normalerweise nicht aufzufinden sind, und eine Immun‑/Entzündungsreaktion auslösen, die viele chronische Entzündungskrankheiten auslösen oder verschlimmern kann [14].

Ein direktes Beispiel für chemische Zytotoxizität ist die lokale Entzündung der Epithelzellen bei Personen mit undichter Epithelbarriere, die als „Epithelitis“ bezeichnet wird. Die Epithelitis ist das erste Ereignis, das proinflammatorische Zellen in den Bereich der geschädigten Epithelbarriere lockt. Sie beginnt mit Umweltbelastungen (Schadstoffe und toxische Substanzen), viralen Infektionen und Enzymen in Allergenen. Vor allem die Alarmine IL-25, IL-33 und TSLP sowie zahlreiche proinflammatorische Chemokine werden von den Epithelzellen freigesetzt, die das Immunsystem in dem Gebiet aktivieren, insbesondere zu Typ-IVb-vermittelten Reaktionen über T2-assoziierte Effektorzellen [49].

Mikrobielle Dysbiose findet in Bereichen mit undichter Epithelbarriere und Entzündungen statt. Eine gesunde Mikrobiota an der Oberfläche der Schleimhautbarriere reguliert zahlreiche Aspekte der Barrierenhomöostase. Eine verminderte Artenvielfalt und Veränderungen in der Zusammensetzung der Mikrobiota von Darm und Haut werden jedoch mit verschiedenen entzündlichen Erkrankungen in Verbindung gebracht, darunter Asthma, allergische Erkrankungen, entzündliche Darmerkrankungen, Typ-1-Diabetes und Adipositas [33]. Dysbiose bezieht sich auf ein Ungleichgewicht der Mikroorganismen, die sich in unseren Geweben befinden, wobei mikrobielle Dysbiose und bakterielle Translokation mit der Entwicklung und Verschlimmerung von allergischen und Autoimmunerkrankungen in Verbindung gebracht werden [62]. Auf die mikrobielle Dysbiose und die Translokation von Kommensalen und opportunistischen Krankheitserregern über die Epithelbarriere folgt in der Regel eine Typ-2-Immunantwort, die durch eine Dominanz von Th2-Zellen, ILC2 und Eosinophilen gekennzeichnet ist. Mastzellen, Makrophagen und Antikörper produzierende B‑Zellen können ebenfalls an dieser Reaktion beteiligt sein. Das Epithel kann die Barriere nicht vollständig reparieren und schließen, wodurch ein Teufelskreis aus undichter Barriere, mikrobieller Dysbiose und chronischer Entzündung in Gang gesetzt wird.

## Diagnostische und therapeutische Implikationen

Die Klassifizierung der CRS und CRSwNP nach immunologischen Endotypen findet immer mehr Eingang in spezifische Krankheitsdefinitionen. Am häufigsten wird in Europa der Typ IVb (ehemals T2-Endotyp) angegeben, den wir in Teil 1 dieser Publikationsreihe dargestellt haben [26].

Die hier vorgestellte Nomenklatur basiert auf Krankheitsmechanismen und Endotypen anstelle von Phänotypen und kann zur Entwicklung neuer Diagnoseinstrumente, verbesserter therapeutischer Strategien und einem besseren Krankheitsmanagement führen. Die epitheliale Barrierestörung spielt eine zentrale Rolle in der Pathophysiologie von chronischer Rhinosinusitis mit Nasenpolypen (CRSwNP) und Asthma.

Eine intakte Schleimhautbarriere ist für die Aufrechterhaltung der Gewebehomöostase von entscheidender Bedeutung, da sie die Nasenschleimhaut vor Infektionen, Umweltgiften, Schadstoffen und Allergenen schützt [1]. Eine gestörte Epithelbarriere wurde bei allergischen und Autoimmunerkrankungen wie allergischer Rhinitis und chronischer Rhinosinusitis (CRS), aber auch bei vergleichbaren Erkrankungen wie Neurodermitis, Asthma, eosinophiler Ösophagitis, Zöliakie und entzündlichen Darmerkrankungen nachgewiesen [1]. Einige Autoren vermuten sogar, dass die Zunahme der die epitheliale Barriere schädigenden Stoffe im Zusammenhang mit der Industrialisierung, Urbanisierung und dem modernen Leben, dem Anstieg von allergischen, autoimmunen und anderen chronischen Schleimhauterkrankungen zugrunde liegt [1]. Es scheint erwiesen zu sein, dass neben der Epithelbarriere ein intaktes Schleimhaut-Immunsystem Voraussetzung für eine Vermeidung chronisch-entzündlicher Schleimhauterkrankungen ist und mukosale Immunstörungen an der Entstehung auch von Krankheiten wie der AR und CRS beteiligt sind [1, 29, 30, 32].

Thymisches stromales Lymphopoietin (TSLP) ist ein hauptsächlich vom Atemwegsepithel exprimiertes Zytokin, das als Reaktion auf Umwelteinflüsse freigesetzt wird und Entzündungsprozesse auslöst [17]. Seine Expression ist bei Asthmapatienten erhöht, korreliert mit Krankheitsschwere und Lungenfunktion, und genetische Polymorphismen werden mit Asthma assoziiert. TSLP gilt somit als zentraler Faktor chronisch-entzündlicher Atemwegserkrankungen, und klinische Studien zur TSLP-Blockade mit monoklonalen Antikörpern waren sehr erfolgreich [17].

Nicht-T2-Entzündungen bei CRS werden häufig durch Th17-Zellen und Neutrophile vermittelt. IL-17A, das von Th17-Zellen produziert wird, induziert in Epithelzellen die Bildung neutrophilenrekrutierender Zytokine wie CXCL8 (IL-8) und GM-CSF [21] und fördert den strukturellen Umbau der Atemwege [7]. TSLP verstärkt die durch Toll-like-Rezeptor-3(TLR3)-Liganden ausgelöste IL-23-Produktion dendritischer Zellen und fördert die Differenzierung naiver CD4⁺-T-Zellen zu Th17-Zellen [55]. Zudem induziert es eine kombinierte Th2-/Th17-Polarisierung mit erhöhter IL-4/IL-17A-Expression und verstärkter Ausschüttung proinflammatorischer Mediatoren wie IL‑6 und IL-23 [35]. Diese Mechanismen zeigen, dass TSLP die Th17-Antwort unter Th2-polarisierten Bedingungen über dendritische Zellen fördert.

Darüber hinaus kann TSLP Neutrophile aktivieren, indem es in das C5-Komplementsystem eingreift und die Bildung reaktiver Sauerstoffspezies moduliert [61]. In chronisch-entzündlichen Atemwegen sind IL-33- und TSLP-Spiegel, nicht jedoch IL-25, erhöht [34]; Neutrophile stellen dabei selbst eine Quelle von TSLP dar [60, 65, 66]. Bemerkenswert ist, dass eine Anti-TSLP-Antikörpertherapie Exazerbationen bei schwerem Asthma mit Nicht-T2-Inflammation reduzierte, was auf eine Rolle von TSLP auch bei nichteosinophilen Entzündungen hinweist [10]. Sein vermehrtes Auftreten etwa bei COPD [65] spricht ebenfalls für eine Beteiligung an T2-unabhängigen Entzündungswegen.

Neben diesen immunologischen Effekten fungiert TSLP als Vermittler zwischen Immun- und Epithelzellen. Eine epitheliale Dysregulation führt zu „airway remodelling“ mit Verdickung der Basalmembran, Becherzellhyperplasie, subepithelialer Fibrose und Hyperplasie oder Hypertrophie glatter Muskelzellen [15]. Die TSLP-Blockade stellt daher einen vielversprechenden Therapieansatz sowohl für T2- als auch nicht-T2-assoziierte Asthmaformen dar und könnte erstmals eine monoklonale Antikörpertherapie für Patienten mit Nicht-T2-Entzündung ermöglichen.

In einer früheren Phase-III-Studie mit Erwachsenen mit schwerem, unkontrolliertem Asthma zeigte Tezepelumab bei Patienten mit zusätzlicher Nasenpolypenanamnese signifikante Verbesserungen des SNOT-22-Scores [38].

Kürzlich wurden die Ergebnisse einer weiteren Phase-III-Studie mit 408 Erwachsenen mit schwerer, unkontrollierter CRSwNP veröffentlicht [36]. Diese multizentrische Studie wurde an 112 Standorten in 10 Ländern durchgeführt; 60 % der Teilnehmenden litten an Asthma, 70 % hatten mindestens eine Nasennebenhöhlenoperation hinter sich, und 17 % wiesen eine Analgetika-Intoleranz auf. Trotz standardisierter intranasaler Kortikosteroidtherapie wurden sie während einer Baseline-Phase eingeschlossen und im Verhältnis 1:1 randomisiert, um 52 Wochen lang alle vier Wochen Tezepelumab (210 mg) oder Placebo subkutan zu erhalten. Beide koprimären Endpunkte – Nasenpolypen-Score (NPS) und Nasenverstopfungs-Score (NCS) in Woche 52 – wurden hochsignifikant erreicht (Effektgrößen: −2,07 bzw. −1,03), mit frühen Verbesserungen nach zwei bis vier Wochen. Sekundäre Endpunkte bestätigten die Überlegenheit von Tezepelumab: Der SNOT-22 verbesserte sich um 27,3 Punkte, der subjektive Geruchsverlust um 1,0 Punkt; nur ein Patient benötigte eine erneute Operation gegenüber 42 in der Placebogruppe. Der Bedarf an systemischen Kortikosteroiden war geringer (5 vs. 36 Fälle), die UPSIT-bewertete Geruchsfunktion und der Lund-Mackay-CT-Score verbesserten sich signifikant. Das Sicherheitsprofil entsprach dem der Placebogruppe. Eine Subgruppenanalyse deutet zudem auf Wirksamkeit auch bei Patienten mit weniger ausgeprägter Typ-2-Entzündung hin.

Wie bei anderen Biologika wird es wichtig sein Biomarker zu finden, die Patienten mit guten Aussichten auf Erfolg einer anti-TSLP-Therapie identifizieren. Eosinophile im Blut, Serum-IgE und FeNO wurden als Biomarker verwendet, um die Behandlung mit monoklonalen Anti-IL-5/IL-5Rα‑, Anti-IgE- und Anti-IL-4/IL-13-Antikörpern zu analysieren. TSLP selbst könnte zwar hypothetisch als Biomarker verwendet werden, doch hat sich dies nicht bewährt, vor allem wegen der Schwierigkeiten bei der Messung niedriger Konzentrationen dieses Zytokins [17].

Darüber hinaus wurde gezeigt, dass TSLP im Nasenpolypengewebe durch endogene Proteasen gespalten wird und hieraus bioaktive Peptide entstehen [41, 46], die bei Messungen mit Anti-TSLP-Antikörper bei Ex-vivo-Untersuchungen nicht nachgewiesen werden können, wodurch die tatsächliche TSLP-Produktion möglicherweise unterschätzt wird [17]. Versuche, TSLP in Patientenproben zu quantifizieren, werden auch dadurch erschwert, dass es zwei Varianten des Proteins gibt: eine Langform-TSLP (lfTSLP), bei der das Protein in voller Länge vorliegt, und eine Form, die etwa die Hälfte der Aminosäurelänge (63 Aminosäuren) umfasst und oft als Kurzform-TSLP (sfTSLP) bezeichnet wird [19, 57, 58, 63]. Weitere Forschung ist erforderlich, um die Rolle der beiden TSLP-Varianten, ihre Regulierung durch SNPs und ihre Expression unter verschiedenen pathologischen Bedingungen besser zu verstehen.

Obwohl TSLP in erster Linie von Epithelzellen an den Barriereoberflächen (Nase, Nasennebenhöhle, Lunge, Darm, Haut) exprimiert wird, kann TSLP auch von einer Reihe von Immunzellen produziert werden. Die systemische Verabreichung von Anti-TSLP birgt daher auch das Potenzial, andere homöostatische Funktionen von TSLP zu stören [12, 16].

## Fazit für die Praxis

- Die chronische Rhinosinusitis, insbesondere die CRSwNP, ist eine heterogene Erkrankung mit unterschiedlichen immunologischen Endotypen, bei der epithelialen Barrieredefekten eine zentrale pathophysiologische Bedeutung zukommt.
- Eine gestörte Schleimhautbarriere fördert die Aktivierung von Alarminen wie TSLP, IL-33 und IL-25 und trägt damit wesentlich zur Chronifizierung der Entzündung bei.
- TSLP fungiert als Schlüsselmolekül zwischen Epithel- und Immunzellen und ist sowohl in T2- als auch Nicht-T2-Entzündungen involviert.
- Die Blockade von TSLP zeigt in klinischen Studien ein vielversprechendes therapeutisches Potenzial bei CRSwNP, auch jenseits klassischer T2-Endotypen.
- Künftig werden Biomarker und ein besseres Verständnis epithelialer und immunologischer Interaktionen entscheidend sein, um gezielte und individualisierte Therapien zu ermöglichen.

## Abkürzungen

AD: Atopische Dermatitis
APC: Antigenpräsentierende Zellen
AR: Allergische Rhinitis
ARC: Allergische Rhinokonjunktivitis
ASMC: Glatte Muskelzellen der Atemwege („airway smooth muscle cell“)
CRS: Chronische Rhinosinusitis
CRSsNP: Chronische Rhinosinusitis ohne Nasenpolypen
CRSwNP: Chronische Rhinosinusitis mit Nasenpolypen
DC: Dendritische Zellen
EAACI: Europäische Akademie für Allergie und klinische Immunologie
ECP: Eosinophiles kationisches Protein
HLA: Humanes Leukozytenantigen
IFN: Interferon
Ig: Immunglobulin
IL: Interleukin
ROS: Reaktive Sauerstoffspezies
SNP: Einzelnukleotid-Polymorphismus („single-nucleotide polymorphism“)
Th: T‑Helfer-Lymphozyten
TNF: Tumornekrosefaktor
TSLP: Thymisches stromales Lymphopoietin

## References

[1] Akdis CA (2021) Does the epithelial barrier hypothesis explain the increase in allergy, autoimmunity and other chronic conditions? Nat Rev Immunol 21:739–751 33846604 10.1038/s41577-021-00538-7

[2] Akdis CA, Bachert C, Cingi C et al (2013) Endotypes and phenotypes of chronic rhinosinusitis: a PRACTALL document of the European Academy of Allergy and Clinical Immunology and the American Academy of Allergy, Asthma & Immunology. J Allergy Clin Immunol 131:1479–1490 23587334 10.1016/j.jaci.2013.02.036 PMC4161279

[3] Bergmann K‑C (2022) Biology of house dust mites and storage mites. Allergo J Int 31:272–278

[4] Bergmann K‑C (2022) Frequency of sensitizations and allergies to house dust mites. Allergo J Int 31:279–283

[5] Brehler R (2023) Clinic and diagnostics of house dust mite allergy. Allergo J Int 32:1–4

[6] Brehler R, Klimek L (2023) Allergen characteristics, quality, major allergen content and galenics for mite allergen-specific immunotherapy preparations. Allergo J Int 32:5–9

[7] Chesné J, Braza F, Mahay G et al (2014) IL-17 in severe asthma. Where do we stand? Am J Respir Crit Care Med 190:1094–1101 25162311 10.1164/rccm.201405-0859PP

[8] Ciprandi G, Schiavetti I, Ricciardolo FLM (2023) Patients with asthma consulting an allergist differ from those consulting a pulmonologist. Allergo J Int

[9] Coombs PRa GPG (1968) Classification of Allergic Reactions Responsible for Clinical Hypersensitivity and Disease. In: Gell RR (Hrsg) Clinical Aspects of Immunology. Oxford University Press, Oxford, S 575–596

[10] Corren J, Parnes JR, Wang L et al (2017) Tezepelumab in Adults with Uncontrolled Asthma. N Engl J Med 377:936–946 28877011 10.1056/NEJMoa1704064

[11] Cuevas M, Polk M‑L, Becker S et al (2022) Rhinitis allergica in storage mite allergy. Allergo J Int 31:59–68

[12] Demehri S, Turkoz A, Manivasagam S et al (2012) Elevated epidermal thymic stromal lymphopoietin levels establish an antitumor environment in the skin. Cancer Cell 22:494–505 23079659 10.1016/j.ccr.2012.08.017 PMC3480666

[13] Desai MS, Anna MS, Arnaud M et al (2016) A Dietary Fiber-Deprived Gut Microbiota Degrades the Colonic Mucus Barrier and Enhances Pathogen Susceptibility. Cell 167:1339–1353.e1321 27863247 10.1016/j.cell.2016.10.043 PMC5131798

[14] Doyle AD, Masuda MY, Pyon GC et al (2023) Detergent exposure induces epithelial barrier dysfunction and eosinophilic inflammation in the esophagus. Allergy 78(1):192–201 35899466 10.1111/all.15457 PMC9797443

[15] Fehrenbach H, Wagner C, Wegmann M (2017) Airway remodeling in asthma: what really matters. Cell Tissue Res 367:551–569 28190087 10.1007/s00441-016-2566-8 PMC5320023

[16] Fornasa G, Tsilingiri K, Caprioli F et al (2015) Dichotomy of short and long thymic stromal lymphopoietin isoforms in inflammatory disorders of the bowel and skin. J Allergy Clin Immunol 136:413–422 26014813 10.1016/j.jaci.2015.04.011 PMC4534776

[17] Gauvreau GM, Sehmi R, Ambrose CS et al (2020) Thymic stromal lymphopoietin: its role and potential as a therapeutic target in asthma. Expert Opin Ther Targets 24:777–792 32567399 10.1080/14728222.2020.1783242

[18] Grosse-Kathoefer S, Aglas L, Ferreira F et al (2023) What inhalant allergens can do and not do?—The cooperation of allergens and their source in Th2 polarization and allergic sensitization. Allergo J Int 32:258–268

[19] Harada M, Hirota T, Jodo AI et al (2009) Functional analysis of the thymic stromal lymphopoietin variants in human bronchial epithelial cells. Am J Respir Cell Mol Biol 40:368–374 18787178 10.1165/rcmb.2008-0041OC

[20] Hiraishi Y, Yamaguchi S, Yoshizaki T et al (2018) IL-33, IL-25 and TSLP contribute to development of fungal-associated protease-induced innate-type airway inflammation. Sci Rep 8:18052 30575775 10.1038/s41598-018-36440-x PMC6303299

[21] Jones CE, Chan K (2002) Interleukin-17 stimulates the expression of interleukin‑8, growth-related oncogene-alpha, and granulocyte-colony-stimulating factor by human airway epithelial cells. Am J Respir Cell Mol Biol 26:748–753 12034575 10.1165/ajrcmb.26.6.4757

[22] Jutel M (2024) Neue Nomenklatur allergischer Erkrankungen nach EAACI-Standard: Teil 3: Nomenklatur von Allergien vom Typ 4 – Executive Summary eines EAACI-Positionspapiers. Allergo J 33:16–27

[23] Jutel M, Agache I, Zemelka-Wiacek M et al (2023) Nomenclature of allergic diseases and hypersensitivity reactions: Adapted to modern needs: An EAACI position paper. Allergy 78(11):2851–2874 37814905 10.1111/all.15889

[24] Jutel MAI, Ollert M, Vieths S, Schwarze J, Akdis Ca, Klimek L (2024) Neue Nomenklatur allergischer Erkrankungen nach EAACI-Standard: Teil 2: Nomenklatur von Allergien vom Typ 2 und 3 – Executive Summary eines EAACI-Positionspapiers. Allergo J 33:16–24

[25] Jutel M, Ollert M, Vieths S et al (2024) Neue Nomenklatur allergischer Erkrankungen nach EAACI-Standard: Teil 1: Grundlagen und Nomenklatur von Soforttyp-Allergien (Typ 1) – Executive Summary eines EAACI-Positionspapiers. Allergo J 33:16–25

[26] Klimek L, Becker S, Haxel B et al. (2025) Verschiedene immunologische Typen der CRSwNP im Kontext der neuen Europäischen EAACI-Nomenklatur : Teil 1: Hypersensitivitätsreaktionen vom Typ IVa–c als Korrelat zu T1-, T2- und T3-Endotypen. Hno 10.1007/s00106-025-01600-9 PMC12185562 40198352

[27] Klimek L, Brehler R, Bergmann K‑C et al (2023) Avoidance measures for mite allergy—an update. Allergo J Int 32:18–27

[28] Klimek L, Brehler R, Casper I et al (2023) Allergen immunotherapy in house dust mite-associated allergic rhinitis: efficacy of the 300 IR mite tablet. Allergo J Int 32:10–17

[29] Klimek L, Casper I, Siemer S et al (2019) T‑cell immune responses in chronic inflammatory diseases of the nasal mucosa. HNO 67:881–892 31598772 10.1007/s00106-019-00759-2

[30] Klimek L, Casper I, Wollenberg B et al (2019) Histamine receptors in chronic inflammatory diseases of the nose and paranasal sinuses. HNO 67:389–400 30944947 10.1007/s00106-019-0649-z

[31] Klimek L, Förster-Ruhrmann U, Beule AG et al (2022) Indicating biologics for chronic rhinosinusitis with nasal polyps (CRSwNP). Allergo J Int 31:149–160

[32] Koennecke M, Klimek L, Mullol J et al (2018) Subtyping of polyposis nasi: phenotypes, endotypes and comorbidities. Allergo J Int 27:56–65 29564208 10.1007/s40629-017-0048-5 PMC5842507

[33] Levy M, Kolodziejczyk AA, Thaiss CA et al (2017) Dysbiosis and the immune system. Nat Rev Immunol 17:219–232 28260787 10.1038/nri.2017.7

[34] Li Y, Wang W, Lv Z et al (2018) Elevated Expression of IL-33 and TSLP in the Airways of Human Asthmatics In Vivo: A Potential Biomarker of Severe Refractory Disease. J Immunol (baltimore Md : 1950) 200:2253–2262 10.4049/jimmunol.1701455 29453280

[35] Liang Y, Yu B, Chen J et al (2019) Thymic stromal lymphopoietin epigenetically upregulates Fc receptor γ subunit-related receptors on antigen-presenting cells and induces T(H)2/T(H)17 polarization through dectin‑2. J Allergy Clin Immunol 144:1025–1035.e1027 31251950 10.1016/j.jaci.2019.06.011

[36] Lipworth BJ, Han JK, Desrosiers M et al (2025) Tezepelumab in Adults with Severe Chronic Rhinosinusitis with Nasal Polyps. N Engl J Med 10.1056/NEJMoa2414482 40106374

[37] Luperto P, Masieri S, Cavaliere C et al (2022) Nasal cytology identifies allergic rhinitis phenotypes for managing allergen immunotherapy in clinical practice. Allergo J Int 31:51–55

[38] Menzies-Gow A, Corren J, Bourdin A et al (2021) Tezepelumab in Adults and Adolescents with Severe, Uncontrolled Asthma. N Engl J Med 384:1800–1809 33979488 10.1056/NEJMoa2034975

[39] Moloudizargari M, Moradkhani F, Asghari N et al (2019) NLRP inflammasome as a key role player in the pathogenesis of environmental toxicants. Proc Natl Sci Counc Repub China B 231:116585 10.1016/j.lfs.2019.116585 31226415

[40] Mülleneisen N, Springob M, Salge S et al (2022) The long road from the first symptoms of mite allergy to mite-specific immunotherapy. Allergo J Int 31:284–287

[41] Nagarkar DR, Poposki JA, Tan BK et al (2013) Thymic stromal lymphopoietin activity is increased in nasal polyps of patients with chronic rhinosinusitis. J Allergy Clin Immunol 132:593–600.e512 23688414 10.1016/j.jaci.2013.04.005 PMC3759596

[42] Nakamura M, Kamiya K, Furuhata A et al (2021) S100A7 Co-localization and Up-regulation of Filaggrin in Human Sinonasal Epithelial Cells. Curr Med Sci 41:863–868 34643881 10.1007/s11596-021-2431-1

[43] Papadopoulos NG, Bernstein JA, Demoly P et al (2015) Phenotypes and endotypes of rhinitis and their impact on management: a PRACTALL report. Allergy 70:474–494 25620381 10.1111/all.12573

[44] Papadopoulos NG, Guibas GV (2016) Rhinitis Subtypes, Endotypes, and Definitions. Immunol Allergy Clin North Am 36:215–233 27083098 10.1016/j.iac.2015.12.001 PMC7127043

[45] Philpott CM, Erskine S, Hopkins C et al (2018) Prevalence of asthma, aspirin sensitivity and allergy in chronic rhinosinusitis: data from the UK National Chronic Rhinosinusitis Epidemiology Study. Respir Res 19:129 29945606 10.1186/s12931-018-0823-y PMC6020303

[46] Poposki JA, Klingler AI, Stevens WW et al (2016) Proprotein convertases generate a highly functional heterodimeric form of thymic stromal lymphopoietin in humans. J Allergy Clin Immunol 139:1559–1567.e1558 27744031 10.1016/j.jaci.2016.08.040 PMC5389936

[47] Rothe T (2018) A century of “intrinsic asthma”—a view on the development of phenotyping in asthma in the last 100 years. Allergo J Int 27:215–219

[48] Saito K, Orimo K, Kubo T et al (2023) Laundry detergents and surfactants-induced eosinophilic airway inflammation by increasing IL-33 expression and activating ILC2s. Allergy 78:1878–1892 37163231 10.1111/all.15762

[49] Schleimer RP, Berdnikovs S (2017) Etiology of epithelial barrier dysfunction in patients with type 2 inflammatory diseases. J Allergy Clin Immunol 139:1752–1761 28583447 10.1016/j.jaci.2017.04.010 PMC5753806

[50] Segboer CL, Fokkens WJ, Terreehorst I et al (2018) Endotyping of non-allergic, allergic and mixed rhinitis patients using a broad panel of biomarkers in nasal secretions. PLoS ONE 13:e200366 30048449 10.1371/journal.pone.0200366 PMC6061980

[51] Shamizadeh S, Brockow K, Ring J (2014) Rupatadine: efficacy and safety of a non-sedating antihistamine with PAF-antagonist effects. Allergo J Int 23:87–95 26120520 10.1007/s40629-014-0011-7 PMC4479428

[52] Soyka MB, Wawrzyniak P, Eiwegger T et al (2012) Defective epithelial barrier in chronic rhinosinusitis: the regulation of tight junctions by IFN‑γ and IL‑4. J Allergy Clin Immunol 130:1087–1096.e1010 22840853 10.1016/j.jaci.2012.05.052

[53] Steelant B, Farré R, Wawrzyniak P et al (2016) Impaired barrier function in patients with house dust mite-induced allergic rhinitis is accompanied by decreased occludin and zonula occludens‑1 expression. J Allergy Clin Immunol 137:1043–1053.e1045 26846377 10.1016/j.jaci.2015.10.050

[54] Sugita K, Steer CA, Martinez-Gonzalez I et al (2018) Type 2 innate lymphoid cells disrupt bronchial epithelial barrier integrity by targeting tight junctions through IL-13 in asthmatic patients. J Allergy Clin Immunol 141:300–310.e311 28392332 10.1016/j.jaci.2017.02.038

[55] Tanaka J, Watanabe N, Kido M et al (2008) Human TSLP and TLR3 ligands promote differentiation of Th17 cells with a central memory phenotype under Th2-polarizing conditions. Clin Exp Allergy 39:89–100 19055649 10.1111/j.1365-2222.2008.03151.x PMC7164823

[56] Uzbekov R, Bouakaz A, Postema M (2023) Closeups of a not-so-domestic mite tritonymph. Allergo J Int 32:337–339

[57] Varricchi G, Pecoraro A, Marone G et al (2018) Thymic Stromal Lymphopoietin Isoforms, Inflammatory Disorders, and Cancer. Front Immunol 9:1595 30057581 10.3389/fimmu.2018.01595 PMC6053489

[58] Verstraete K, Peelman F, Braun H et al (2017) Structure and antagonism of the receptor complex mediated by human TSLP in allergy and asthma. Nat Commun 8:14937 28368013 10.1038/ncomms14937 PMC5382266

[59] Vrtala S (2022) Allergens from house dust and storage mites. Allergo J Int 31:267–271

[60] Wang W, Li Y, Lv Z et al (2018) Bronchial Allergen Challenge of Patients with Atopic Asthma Triggers an Alarmin (IL-33, TSLP, and IL-25) Response in the Airways Epithelium and Submucosa. J Immunol (baltimore Md : 1950) 201:2221–2231 10.4049/jimmunol.1800709 30185520

[61] West EE, Spolski R, Kazemian M et al (2016) A TSLP-complement axis mediates neutrophil killing of methicillin-resistant Staphylococcus aureus. Sci Immunol 1:10.1126/sciimmunol.aaf8471 PMC8530006 28783679

[62] Wood LG (2017) Asthma in the Obese: A Big and Growing Problem. Am J Respir Crit Care Med 195:4–5 28035860 10.1164/rccm.201608-1582ED

[63] Xie Y, Takai T, Chen X et al (2012) Long TSLP transcript expression and release of TSLP induced by TLR ligands and cytokines in human keratinocytes. J Dermatological Sci 66:233–237 10.1016/j.jdermsci.2012.03.007 22520928

[64] Yazici D, Ogulur I, Kucukkase O et al (2022) Epithelial barrier hypothesis and the development of allergic and autoimmune diseases. Allergo J Int 31:91–102

[65] Ying S, O’connor B, Ratoff J et al (2008) Expression and cellular provenance of thymic stromal lymphopoietin and chemokines in patients with severe asthma and chronic obstructive pulmonary disease. J Immunol (baltimore Md : 1950) 181:2790–2798 10.4049/jimmunol.181.4.2790 18684970

[66] Ying S, O’connor B, Ratoff J et al (2005) Thymic stromal lymphopoietin expression is increased in asthmatic airways and correlates with expression of Th2-attracting chemokines and disease severity. J Immunol (baltimore Md : 1950) 174:8183–8190 10.4049/jimmunol.174.12.8183 15944327

[67] Zhu J (2015) T helper 2 (Th2) cell differentiation, type 2 innate lymphoid cell (ILC2) development and regulation of interleukin‑4 (IL-4) and IL-13 production. Cytokine 75:14–24 26044597 10.1016/j.cyto.2015.05.010 PMC4532589

